# The perception of parents and teachers about intermittent preventive treatment for malaria in school children in a semi-rural area of Kinshasa, in the Democratic Republic of Congo

**DOI:** 10.1186/s12936-016-1670-2

**Published:** 2017-01-07

**Authors:** Junior R. Matangila, Jessica Fraeyman, Marie-Louise Mbula Kambulu, Alain Mpanya, Raquel Inocêncio da Luz, Pascal Lutumba, Jean-Pierre Van Geertruyden, Hilde Bastiaens

**Affiliations:** 1 Département de Médecine Tropicale, Université de Kinshasa, Kinshasa, Democratic Republic of the Congo; 2 Epidemiology for Global Health Institute, University of Antwerp, Campus Drie Eiken, Universiteitsplein 1, 2610 Wilrijk, Belgium; 3 Research Group Medical Sociology and Health Policy, University of Antwerp, Antwerp, Belgium; 4 Department of Diseases Control, Ministry of Public Health, Kinshasa, Democratic Republic of the Congo; 5 Department of Primary and Interdisciplinary Care, University of Antwerp, Antwerp, Belgium

## Abstract

**Background:**

Intermittent preventive treatment (IPT) is likely to be the most promising therapeutic strategy to prevent malaria and its related adverse outcomes in schoolchildren. However, its successful implementation will depend on acceptability to key stakeholders such as parents and teachers.

**Methods:**

A qualitative research was conducted, following a clinical trial assessing the effectiveness of IPT in schoolchildren (IPTsc), to understand the perceptions and experiences of parents and teachers with IPTsc, in two schools of Mokali, in Kinshasa, Democratic Republic of the Congo. Eighty parents participated in 8 focus group discussions and 6 school staff were involved in 6 semi-structured interviews.

**Results:**

Parents experiences with IPTsc divided them into two groups (owning positive experiences and owning negative experiences with IPTsc). Three major themes emerged as key factors associated with reluctance of parents to IPT use in schoolchildren. These included wrong malaria-related knowledge, bad experience with IPTsc administered during the trial and misunderstanding of IPTsc. The school staff were generally willing to be trained to give medicine to schoolchildren within the scope of IPT. However, most parents were more comfortable with the use of health workers than teachers for drug administration. More importantly, all parents accepting IPT suggested to diagnose malaria infection before any administration of IPT, which is not in line with IPT principal.

**Conclusion:**

These results suggest that more efforts are needed to improve overall malaria-related knowledge in the community, specifically chemo-prevention strategies and the safety of the drugs used, to ensure the success of health interventions.

## Background

Malaria is a major parasitic disease in developing countries and particularly sub-Saharan Africa (SSA). In human, malaria is caused in by five Plasmodium parasites, the most severe being *Plasmodium falciparum* [1]. More than half of the world population lives in malaria endemic areas. Globally, 214 million clinical malaria cases and 438,000 deaths have been reported in 2015. Most deaths (90%) occurred in African region [2].

At present, the development and implementation of interventions for prevention and treatment of malaria focuses on the most vulnerable population groups, *in casu* pregnant women and children less than 5 years [3]. However, malaria remains a major cause of mortality and morbidity among school children and can have profound consequences on learning and school performances and general development [4, 5]. There is a growing recognition by many African governments of the importance of child health for educational achievements [6], and an increasing number of low-income countries now use their schools as platforms for delivering simple, safe and cost-effective health and nutrition interventions, such as deworming [7]. However, while there is growing awareness of the importance of reducing the burden of malaria in school children and political support for school-based malaria control, there is still limited evidence to guide the formulation of policy [8, 9]. Intermittent preventive treatment (IPT) is likely to be the most promising therapeutic strategy to prevent malaria and its related adverse outcomes in school-aged children. IPT treats patients at long intervals, permitting drug concentrations to fall below the minimal inhibitory concentration (MIC) between treatment courses, this regardless of whether they have malaria infection or not. To date only few studies have been conducted on intermittent preventive treatment in schoolchildren (IPTsc), providing little evidence on the more appropriate drug regimen to be used and the acceptability of this strategy to key stakeholders such as parents, teachers and local implementers [10]. This underlines the need to conduct studies to ascertain the adapted drug regimen for IPTsc and to explore IPTsc acceptability for a successful implementation. In this perspective, a randomized controlled trial (RCT) investigating the impact of ITP with sulfadoxine–pyrimethamine (SP) and SP plus piperaquine (SP+PQ) on anaemia and malaria morbidity in schoolchildren was conducted in the Democratic Republic of Congo (DRC) [11]. DRC is one of the most affected countries by malaria and this study revealed that a high proportion of parents or legal tutors (32.8%) prevented their children from participating in the trial. This high refusal rate may, indirectly, suggest the existence of hindering factors to IPT in schoolchildren (IPTsc) or other malaria interventions in the school community (parents and school staff). The present qualitative study aimed to describe understanding perceptions and experiences relating to IPTsc of the school community selected for this RCT and identify and understand factors including socio-cultural, national and regional factors that may affect acceptance of IPTsc.

## Methods

### Study design

This was a partially mixed sequential design [12] with a main RCT study and a qualitative research study conducted one year after the main study.

#### Main study: randomized controlled trial

The study was an unblinded RCT enrolling asymptomatic school children of either gender. Within each school, each schoolchild was randomly assigned to one of the two intervention groups or the control group as follows: IPT-SP, IPT-SP+PQ and no-treatment. Each study arm consists of one-third of all individuals. At baseline, month four, and month seven, IPT with SP or SP+PQ was given. SP was administered as a single treatment dose. However, PQ was given as two doses with a 24-h interval. IPT was administered at 4 month intervals in line with the long half-lives of the drugs and for compliance. Due to the high prevalence of soil transmitted helminths (STH) and schistosomiasis in the study area, all participants were treated with albendazole or praziquantel according to the WHO guidelines [11]. Before the study, a series of meetings were also held with school staff and parents or legal tutors, in the selected schools to explain the nature and purpose of the trial. Written informed consent was obtained from each parent or legal guardian of all children prior to enrolment. An oral assent was obtained, as well, from children who were 12-years-old or older. The trial design was fully described elsewhere [11].

#### Qualitative research study

A descriptive qualitative approach [13] involving parents/legal tutors and school staff (teachers and directors) was used to explore the understanding, perceptions and experiences on/with IPTsc for malaria.

### Study participants and sampling procedure

Participants were parents or legal tutors of children from the two selected schools for the clinical trial. Parents/legal tutors were sent an written invitation and contacted by phone call to participate in the qualitative study, using a list of names of parents whose children were invited to participate in the trial, this included those whose children were enrolled in the clinical trial, those who did not allow their children to participate in the study and those who consented but whose children did not meet the trial inclusion criteria. However, the constitution of groups was done purposively, according to parents or legal tutors availability and willingness to participate to the qualitative study, regardless of parents’ gender and opinion about IPTsc within schools, as this would be an ideal realm for sharing opinion between parents. Another reason of mixing group was also to avoid stigmatizing non-consenting parents by putting them in a specific group. Teachers from the two selected schools also participated in this qualitative study. Parents/legal tutors and teachers were included in study until saturation of the explored themes was reached. This saturation was reached when no new ideas or information emerge from subsequent transcripts.

### Study site

The studies took place in the Mokali health area of the Biyela health zone, in Kinshasa province, in DRC. Mokali has an estimated 27,455 inhabitants. École Primaire (EP) Boyambi and EP Likabo, two of the nearest primary schools to the regional health centre of Mokali were selected for the trial. These schools were built by the Catholic Church and each school has 14 teachers and 12 classes (two classes per each school grade). The estimated number of schoolchildren was 600 per school at the beginning of the school year in September 2014.

### Data collection

Data were collected from December 2014 till January 2015, more as one year after the trial data collection stopped. During these two months, two trained field workers fluent in the local language (Lingala) collected the qualitative data. Participants were initially contacted by telephone to identify a suitable moment for the interview. Focus group discussions (FGDs) were used to understand parents and legal tutors perceptions of the malaria-related problem in school children, malaria testing and treatment, knowledge and experiences with IPTsc in school children. Semi-structured interviews (IDIs) were conducted with teachers of whom two were also directors. both FGDs and IDIs were audiotaped and pre-tested and semi-structured topic guides that focused on respondents’ malaria related understanding and experiences of the trial procedures, IPTsc and their ideas of medication delivery using teachers.

These guides were pretested in an another semi-rural area, involving parents of schoolchildren. Before the pre-test, explanation on IPTsc and a possible trial to be conducted were given to parents, as their awareness on IPTsc should be as close as possible to that of parents, after the pre-RCT meeting. To ensure rigor and quality in the process of transcribing, trained field staff conducted transcription and translation under the supervision of the principal investigator (PI), who checked for errors. All FGDs and interviews were translated into French. Quotes selected for the article were translated into English by the PI (JM).

### Data analysis

The data from the interviews and FGDs was analysed thematically. Therefore, the authors approached the data transcripts in an inductive approach, marking the pieces of text that provided answers to the research question (referred to as open codes). These open codes were then grouped into themes that overarch broader ideas that came out of the interviews and FGDs. These themes were reorganized into a codebook which was subsequently used to analyse all interviews and FGDs and as such provides an overview of the overall results of the qualitative study. The codebook is supported by a conceptual diagram that illustrates the relationships between the different themes. The PI used QSR NVivo 10 software as an assistant tool to facilitate the analysis.

## Results

A total of eight FGDs, involving 80 parents of schoolchildren, and 6 IDIs with teachers and directors were conducted. A number of key themes that may influence the acceptability of IPTsc were identified: parents understanding of malaria, their experiences with and trust in IPTsc, and their ideas on the role that teachers can play in the programme. These elements influence each other and could negatively influence the acceptability of the programme, as will be described below. Finally, parents also gave suggestions to improve acceptability of the IPTsc programme. The last part of the results describe the perceptions of the school teachers.

### Parents understanding about cause of malaria, symptoms, consequences and preventive measures in school children

Nearly all participants indicated that malaria was caused by mosquitos often found in dirty and wet environments, mainly in the dry season. However, some of them mentioned that malaria was a dirty hands spread disease or caused by drinking not treated or unfiltered water.

“Malaria is an infection that comes from mosquitoes; depending on season, especially when it’s cold during the dry season.” (Parent FGD 2)

“Malaria is a dirty hands spread disease.” (Parent FGD 3)

“Malaria is sometimes caused by drinking not treated or unfiltered water” (Parent FGD 3);

Fever persisting 2 or 3 days, fatigue, headache, and generally child not playing were reported by the majority of the FGD participants to be prominent malaria-related features in schoolchildren. Other conditions associated with malaria in schoolchildren were shivering and coldness.

All FGDs participants recognized that malaria has adverse effects on the health of schoolchildren. These included anaemia, death, school absenteeism, convulsions, poor school performances. However, some participants believed that malaria leads to meningitis, epilepsy, madness in adulthood, sleeping sickness, behavioural disorders, stubbornness or nervousness.

“Mostly during rainy season, malaria causes school absenteeism”

“After a while you hear that the child haemoglobin has decreased due to malaria, and this is especially one of the causes of child deaths.” (Parent FGD 2)

“After the child has suffered from malaria, it leaves him with a depression of intelligence, he will no longer be intelligent.” (Parent FGD 1)

“Some children become stubborn after suffering from malaria, some become so nervous even for a small problem, some children become very rude if they have been heavily affected by malaria.” (Parent FGD 1)

“untreated malaria in children can lead to convulsions and meningitis” (Parent FGD 3)

“Some children suffering for malaria may develop sleeping sickness or madness in they adulthood” (Parent FGD 5)

Parents reported the use of the following strategies to protect their schoolchildren against malaria: administration of anti-malaria drug every 2–3 months, use of traditional plant medicines and use of bed nets. Other reported approaches were avoiding mosquitos by filling holes, improving cleanliness, removing used tins and bags, avoiding stagnant water, closing windows before 18 h. One parent mentioned drinking filtered water as a mean to prevent malaria.

“I often given traditional treatment against malaria to my children, I boil some herbs I give them to drink.” (Parent FGD 2)

“I give a traditional treatment to my children once every two months, as product is bitter they take it with difficulty.” (Parent FGD 8)

“I often use lemongrass for malaria prevention as I had read in a book that this has antimalarial properties.” (Parent FGD 8)

“In my home, necessarily everyone has to sleep under the bed net, whether it is hot or not; I watch myself, if in one room the bed net is not in place I install it. If I realize that it has holes or it’s damaged, I replace it.” (Parent FGD 3)

### Parents perception and experiences with intermittent preventive treatment in school children

Opinions about the IPTsc varied widely. Some reports were positive: IPTsc is a good strategy for malaria prevention for schoolchildren, it is helpful and should continue, it protects against severe form of malaria. This is related to own positive experiences with IPTsc (themselves, own children, children form other parents) and to a positive attitude towards IPTsc at school (it reaches everyone, medication works).

“It’s good and it will also facilitate the task to those who come administer these products because all children do not live in one place, there are some who come from very far away, as Famua and Kikimi then it will be difficult to give them these drugs at home, it’s better when they are all in school where it can be done easily.” (Parent FGD 8)

“We have a serious problem with malaria and your product helped so much here, we ask that it be continued because a sick child cannot study well.” (Parent FGD 2)

“I think it’s good to give preventive treatment medication to children, because when we were young as I said earlier, they were also gave us the dalaprim® at school. This method was successful in the old days, we can come back to it for our children.” (Parent FGD 3)

“If I came here is because I have good memories of preventive treatment you gave to my eldest daughter, who made six whole months without even a slight fever.” (Parent FGD 8)

Negative reports included ideas that the drug used for IPTsc was harmful and exposed children to severe illness and forced parents to spend money for treatment, the drug was not efficacious, the drug was not well administrated, the drug used have the potential to affect children memory and cognitive functions, the drug was expired, the drug could not be given before any laboratory results confirming infection. There were some complaints about lack of information from parents before drug administration to children. One parent mentioned that because of religious belief, he must be present when drug is administered to his child.

“For malaria the laboratory results must confirm the infection before taking any treatment, I cannot agree to take a product or give it to a child without laboratory confirmation.” (Parent FGD 5)

“The drug you used must be expired or toxic; because it is not clear why it was given free of charge and only to health children, without any evidence of infection” (Parent FGD 5)

“My son took the treatment you had administered here after a few days he had high fever, he fell seriously ill, arrived at to anaemia and he even was transfused.” (Parent FGD 4)

“For me, I think that you can change your product because the drug you have given the other time did nothing, the children always fall sick. Look for another product which has the potential to cure completely and definitively malaria.” (Parent FGD 1)

“My little son took that preventive treatment, it was well after a few days, she fell seriously ill. When I got the information that you came again for this IPT, I firmly forbade him you take the drug again it to avoid that I spend money in private hospital for treatment.” (Parent FGD 2)

### Parents perception of the role of teachers to administer IPTsc

Most participants reported that teachers were not qualified to give treatment to schoolchildren. However, very few parents mentioned that teacher could also give treatment as parent do at home for their own children. A number of parents, however, thought that it was safe to train teachers before they perform this task.

“Health workers should give drugs to kids, not untrained people like teachers because in case of an accident parents will not agree. We are glad you thought of preventive treatment but we will accept it as long as only caregiver administer the treatment to our children.” (Parent FGD 8)

“The teachers can always administer medication to the child especially if it is drinkable, as we all do in our homes without nursing staff.” (Parent FGD 4)

“I stand for the teachers involvement because they will always be there with children and whenever there is a something wrong, they will notice that the child is not good. In addition, if teachers selected for this task are trained, I think they will be very much effective than community health workers.” (Parent FGD 8)

### Parents suggestions for effective malaria IPTsc

The majority of parents or guardians suggested that parents must be informed before any drug administration at school, laboratory results should confirm the infection before any IPTsc administration, treatment should be given to all children in the city of Kinshasa and children developing severe illness after IPTsc administration should be treated free of charge.

“parents must be informed prior administration of any drug at school” (Parent FGD 4)

“laboratory analysis should confirm malaria infection before any treatment” (Parent FGD 5)

“children who develop severe sickness after drug administration must receive appropriated health care which must be free of charge”. (Parent FGD 1)

“If you administer these products in this school you do nothing because the malaria circuit will continue. Generalize it to all schools for all children because the future of this country is not limited to these children who study here. It will be better to do it all over Kinshasa.” (Parent FGD 1)

### School staff perception of SHP, malaria, IPTsc and their role in the drug administration

Teachers and directors perceived SHP as a good strategy dedicated to schoolchildren health. Most of them believed that malaria affects negatively schoolchildren’s health. The main reported consequence of malaria was death. The use of bed net and improving cleanliness were the major strategies to prevent malaria in schoolchildren according to school staff. IPTsc was regarded as an important mean to protect schoolchildren against malaria. Teachers also indicated that they would be able to give the drug to schoolchildren if they are well trained. This would be a major condition for parents to allow their children to be treated by teachers.

“When malaria is badly treated, it causes death” (Teacher IDI 1)

“Malaria exposes schoolchildren to many other diseases” (Teacher IDI 2)

“Malaria induced anaemia in schoolchildren” (Teacher IDI 2)

“Malaria kills more than HIV” (Teacher IDI 2)

“to prevent malaria in children we should avoid the dirty waste and use bed net” (Teacher IDI 1)

“the use of drug to prevent malaria symptoms in schoolchildren is a good idea” (Teacher IDI 4)

“Yes, the teacher can give drug to school children it they are trained” (Teacher IDI 2)

## Discussion

This study explores parents or legal tutors’ malaria-related understanding and experience with intermittent preventive treatment in schoolchildren (IPTsc). Based on our data, parents were generally aware that malaria had deleterious effects on schoolchildren health, though their understanding about the malaria transmission and its consequences were incomplete or not always accurate. Malaria prevention (any strategy) was perceived as crucial for their childrens’ health. However, participants’ perceptions and experience with IPTsc differed between parents, splitting them into two groups (willing and not willing to use IPTsc as a means to protect children against malaria).

The most important factor involved in the reluctance to IPTsc was the perceived drug safety. A number of participants believed IPTsc made their children sick, causing additional house-hold expenditures and the drug used was expired or harmful for children brains. However, no serious adverse event was reported during the trial and no drop out was reported to be caused by any adverse event, unless children who had experienced such adverse events were among those lost to follow-up [14]. It appears that some parents associated new strategies (such as IPTsc) to the use of new or unusual drugs. This highlights a poor awareness of the nature of drugs used for IPTsc during the trial, which most probably derived from either poor information delivery during different meetings organized to explain the purpose of the trial. Many parents missed those information sessions and signed whether or not the informed consent, sent to them through their children, without having accurate information about the trial. Issues around informed consent or awareness about trial by enrolled participants, or their parents/legal tutors for children, are common and were also reported by other authors [15]. Beside this, parents were more inclined to use drugs for clinical malaria treatment than for its prevention. Therefore, it was unclear to treat, without any laboratory evidence of infection, an apparently healthy child. As a consequence, all participants who regarded IPTsc as a good mean of prevention suggested that a positive laboratory test for malaria should necessarily precede any preventive treatment, which would turn IPTsc into intermittent screening and treatment (IST) strategy. In this regard, IST is potentially more appealing to parents of schoolchildren than IPTsc. This observation was also reported, in pregnant women, by Pell et al. [16].

Malaria-related understanding in parents and teachers could, to a lesser extent, influence the success of the implementation of IPTsc. In effect, if malaria is known to be caused by drinking unfiltered or not treated water, it is not clear why one would prevent it by administering anti-malarial drug.

Educator (school staff) perception of malaria was roughly similar to that of parents. Moreover, teachers were willing to be trained to give IPTsc to schoolchildren. This study raised an important issue of considering the role of teacher in the delivering system of IPTsc. School community or environment is made up of schoolchildren, parents and staff. Therefore, as a component of this school environment teachers have a key role to play for health promotion [17]. In our conceptual model of the delivering system of IPTsc, one approach stands for the use of teachers to administer IPTsc drug and monitor all related-adverse effects, as during the school years, children spend most of their time with their teachers. However, not all parents agreed with the idea of allowing teachers to give medication to their children. A number of parents/tutors suggested the use of health workers for treatment administration. Those who were comfortable with the involvement of teacher in giving drug, stressed, nevertheless on the principal that these teachers had to be trained before performing this task.

The present study may have some limitations. One major limitation is that this study did not involve community health workers, which could provide a complete picture of the all school community as they are supposed to play a key role for IPTsc administration, in the perspective that teachers may not be eligible.

However, this is to our knowledge the first published study assessing the acceptability of IPTsc in schoolchildren as previously qualitative studies were mainly, directed towards pregnant women and infants who are perceived as at greater risk of malaria. Therefore, our findings could not be compared to those reported by others studies for this specific group of schoolchildren. Nevertheless, although IPT in other groups (pregnant women and infants) is generally accepted, resistance and reluctance is common, even though the reasons behind are not always similar [18, 19].

## Conclusion

Data from this study suggest that most barriers and problems raised from the perceived drug safety and from a general lack of understanding of malaria and principles of IPTsc. Therefore, more efforts are needed to improve overall malaria-related knowledge in the community, with a focus on chemo-prevention strategies and the safety of drug used, to ensure the success of health interventions. Although these are findings from experiences in a controlled trial setting, the study provides an insight into the potential motivating factors and barriers that should be addressed for successful routinely delivering of IPTsc. This study also portrayed the necessity to assess the implementation conditions in order to fully evaluate a more convenient scheme of IPTsc delivery involving trained teacher.

## Abbreviations

DRC: Democratic Republic of the Congo
EP: École Primaire
FGD: focus group discussion
IDI: in-depth interview
IPT: intermittent preventive treatment
IPTsc: intermittent preventive treatment in schoolchildren
MIC: minimal inhibitory concentration
PI: principal investigator
RCT: randomized controlled trial
SP: sulfadoxine–pyrimethamine
SP + PQ: sulfadoxine–pyrimethamine plus piperaquine
SSA: sub-Saharan Africa
SHP: school health programme
WHO: World Health Organization
